# Development and validation of a sunlight exposure questionnaire for urban adult Filipinos

**DOI:** 10.4178/epih.e2018050

**Published:** 2018-10-11

**Authors:** Marc Gregory Yu, Nina Castillo-Carandang, Maria Elinor Grace Sison, Angelique Bea Uy, Katrina Lenora Villarante, Patricia Maningat, Elizabeth Paz-Pacheco, Eileen Abesamis-Cubillan

**Affiliations:** 1 Section of Endocrinology, Diabetes and Metabolism, Department of Medicine, Philippine General Hospital, University of the Philippines, Manila, Philippines; 2 Department of Clinical Epidemiology, University of the Philippines College of Medicine, Manila, Philippines; 3 Institute of Clinical Epidemiology, University of the Philippines-National Institutes of Health, Manila, Philippines; 4 Section of Dermatology, Department of Medicine, Philippine General Hospital, University of the Philippines, Manila, Philippines; 5 Department of Medicine, Philippine General Hospital, University of the Philippines, Manila, Philippines; 6 Department of Family and Community Medicine, Philippine General Hospital, University of the Philippines, Manila, Philippines

**Keywords:** Vitamin D deficiency, Sunlight, Surveys and questionnaires, Philippines

## Abstract

**OBJECTIVES:**

To develop and validate a self-reported sunlight exposure questionnaire (SEQ) for urban adult Filipinos.

**METHODS:**

The study included adults (19-76 years old) in Metro Manila, Philippines, well-versed in the Filipino (Tagalog) language and had resided in Metro Manila for at least 1 year. Exclusion criteria included pregnancy, active skin disorders, and immunocompromised states. An expert panel created a questionnaire in Likert-scale format based on a conceptual framework and 4 existing instruments. The study proceeded in 4 phases: questionnaire item development, translation and back-translation, pretesting, and construct validity and reliability testing using factor analysis, the Cronbach alpha coefficient, and the paired *t*-test.

**RESULTS:**

A 25-item, self-administered, Filipino (Tagalog) SEQ answerable using a 4-point Likert scale was created. The questionnaire was administered to 260 adult participants twice at a 2-week interval, with all participants completing both the first and second rounds of testing. All questionnaire items possessed adequate content validity indices of at least 0.86. After factor analysis, 3 questionnaire domains were identified: intensity of sunlight exposure, factors affecting sunlight exposure, and sun protection practices. Internal consistency was satisfactory for both the overall questionnaire (Cronbach alpha, 0.80) and for each of the domains (Cronbach alpha, 0.74, 0.71, and 0.72, respectively). No statistically significant differences were observed in the responses between the first and second rounds of testing, indicating good test-retest reliability.

**CONCLUSIONS:**

We developed a culturally-appropriate SEQ with sufficient content validity, construct validity, and reliability to assess sunlight exposure among urban adult Filipinos in Metro Manila, Philippines.

## INTRODUCTION

Vitamin D deficiency (VDD) is a major public health concern [1]. It is reflected by low serum 25-hydroxyvitamin D (25-OHD) levels, which lead to adverse changes in calcium and phosphate homeostasis and increased fracture risk. In the Philippines, a 2009 study of postmenopausal women found that 36% of the participants had insufficient 25-OHD levels (20-30 ng/mL), but only 30% of those women received calcium and vitamin D supplementation [2]. Another study of 369 randomly selected Metro Manila office workers in 2014 revealed that 58% of the participants had deficient serum 25-OHD levels (< 20 ng/mL), while 30% had insufficient levels [3].

Exposure to ultraviolet rays (UVB) is the main source of vitamin D in humans. This is because the enteral route is not a good source of vitamin D unless foods are fortified with vitamin D [4]. A contributing factor to the increasing prevalence of VDD in the Philippines is rapid urbanization, which has resulted in more young adults having indoor jobs and thus less sun exposure, raising concerns about bone health during the period when they are achieving peak bone mass. Furthermore, air pollution in major Philippine cities decreases the amount of UVB that reaches the earth’s surface [5].

A major limitation in the area of VDD research is the lack of an appropriate, inexpensive, and easily-administered tool for measuring sunlight exposure [6]. Compared with other methods, questionnaires are considered to be the most cost-effective way of measuring sunlight exposure in population-based studies [7]. Of the available sunlight exposure questionnaires (SEQs), only 2 were validated in Asia (Hong Kong and Pakistan) [6,7] and only 3 were created in the context of VDD by correlating the questionnaire results with serum 25-OHD levels, showing moderate correlations [6,8,9]. At present, there is no existing SEQ that has been validated for Southeast Asian or tropical populations. This study aimed to develop and validate a culturally-appropriate, self-reported SEQ for urban adult Filipinos.

## MATERIALS AND METHODS

### Study participants

The study included individuals >19 years old who were fluent in the Filipino (Tagalog) language, lived in Metro Manila at least 5 days a week for at least 1 year, and provided written informed consent. We set 1 year as the minimum duration of urban living to account for all possible weather changes and seasonal variations. Those who were pregnant or who had known active skin disorders or immunocompromised states potentially affecting sunlight exposure were excluded. Study participants were selected by purposive sampling from any of the 17 component cities of Metro Manila using a sampling frame (Table 1). Based on an existing study on the prevalence of VDD in the Philippines (58% among 369 participants), with confidence limits of 5% and a design effect 2of 1.0, an initial sample size of 187 was calculated [3]. However, after development of the final questionnaire, the sample size was increased to 250 since we took into account the recommended 10:1 subject-to-item ratio for factor analysis [10]. Ten additional subjects were recruited to ensure that participants more equally distributed across the various brackets—15 each for the working-age groups and 10 each for the elderly groups— yielding a final sample size of 260. Epi Info version 7.0 (https://epi-info.software.informer.com/7.0/) was used for sample size calculation.

### Study procedures

The study proceeded in 4 phases.

#### Phase I: questionnaire item development

**Figure 1.**
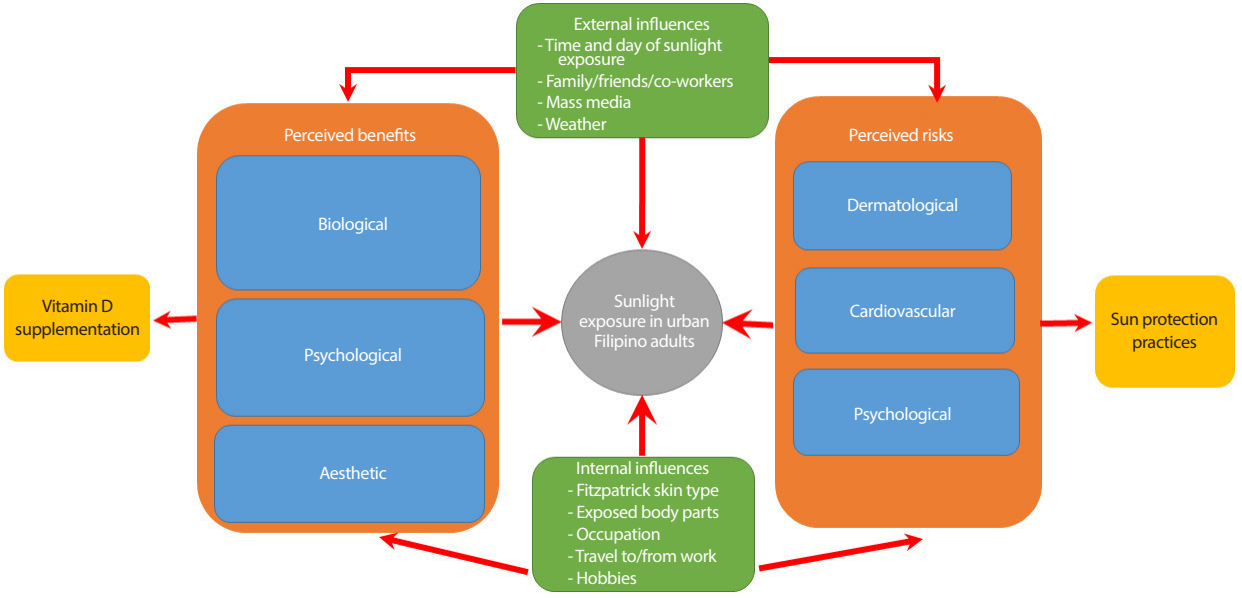
Conceptual framework on the attitudes, behaviors, and beliefs of urban adult Filipinos on sunlight exposure. Adapted from Yu et al. JAFES 2018;33:37-43 [11], on the basis of Open Access.

An extensive literature review of concepts on sunlight exposure assessment was performed and relevant questionnaires were identified, using the main keywords “sunlight,” “questionnaire,” “urban,” “vitamin D,” and “osteoporosis.” There were no restrictions on language, country, or year of publication. Based on their relevance (mainly the inclusion of different sun exposure variables and/or correlation with serum 25-OHD), the SEQs developed by Cargill et al. [9] in Australia, Hanwell et al. [8] in Italy, Humayun et al. [6] in Pakistan, and Wu et al. [7] in Hong Kong were used as references for this study. In addition, an existing conceptual framework on the attitudes, behaviors, and beliefs of urban adult Filipinos on sunlight exposure was also used (Figure 1) [11].

A panel of 3 endocrinologists, 2 dermatologists, a health social scientist, an internist, and a community medicine physician created preliminary questionnaire items using the following guidelines: (1) Is the item unbiased? (2) Is there a strong likelihood that most respondents will answer the item truthfully? (3) Do most respondents possess sufficient knowledge needed to answer the item? (4) Will most respondents be willing to answer the item? (5) Does the item avoid leading respondents to a specific answer? and (6) Is the language used clear and simple enough so that respondents are able to understand all questions? [12].

The questionnaire items were constructed in the form of a 4-point Likert scale and arranged using the following guidelines: (1) Non-sensitive questions were placed at the beginning, since they were assumed to be non-threatening and tended to put the respondent at ease; (2) Items of major interest to the study were also prioritized, since there was greater probability of the respondent completing the first part of the questionnaire; (3) Sensitive items were placed last so that any potential emotions provoked would not influence the responses to other questions; and (4) As much as possible, items on similar topics were placed close to each other [12].

All questionnaire items then underwent content validity assessment by each panel member using a 4-point ordinal scale: 4, very relevant; 3, somewhat relevant; 2, hardly relevant; and 1, not at all relevant. The content validity index (CVI) of each item was computed as the number of evaluators giving a 3 or 4 divided by the total number of evaluators. Only items with a CVI of at least 0.86 were retained in the questionnaire [13].

#### Phase II: forward-translation and back-translation

The items of the draft questionnaire were then translated from English to Filipino (Tagalog) by 2 independent bilingual translators, one of whom was a physician with knowledge of the study and its concepts, and the other a non-medical professional with no knowledge of the study or its concepts. The linguistic and cultural quality of the 2 translations was reviewed individually by the panel experts and was consolidated into a single version by consensus. The newly-synthesized Filipino SEQ then underwent backward translation by a different bilingual translator who was uninvolved in the study. Finally, both translated and back-translated versions were reviewed by the panel experts to reach a consensus for the prefinal Filipino (Tagalog) questionnaire version [14].

#### Phase III: pretest

Pretesting of the prefinal questionnaire to test flow and comprehensibility was performed by administering it to a sample equivalent to thrice the number of questionnaire items. Questionnaires were self-administered and the time needed to complete the test was noted for each person. After answering the questionnaire, the participants were asked for feedback regarding comprehensibility and format using a cognitive debriefing form. The form utilized both close- and open-ended questions. Revision of the questionnaire was then carried out by the panel members based on pretest results and feedback to create the final Filipino version of the SEQ.

#### Phase IV: construct validity test and reliability test

The final questionnaire was administered to the sample population twice at a 2-week interval, with a similar procedure as that of the pretest. A research assistant was accordingly trained in the recruitment of participants and questionnaire administration. Data from the first test underwent factor analysis to determine the different questionnaire domains; items that possessed similar factor loading values were grouped under a particular domain.

Reliability of the questionnaire was assessed by internal consistency and by test-retest reliability. Internal consistency was analyzed using the Cronbach alpha coefficient, with 0.7 as an acceptable cutoff value [15], while test-retest reliability was analyzed using the paired *t*-test. Statistical analyses were performed using Stata SE version 13 (StataCorp., College Station, TX, USA). Descriptive statistics included the mean and standard deviation for normally distributed quantitative variables, median and interquartile range for non-normally distributed quantitative variables, and frequency and percentage for qualitative variables. The threshold for statistical significance was set at 5%.

### Ethics statements

Both the study protocol and informed consent forms were approved by the University of the Philippines institutional review board prior to commencement (UPMREB code: MED-2016-003-01). Study participants also received a token honorarium for their participation.

## RESULTS

Table 2 summarizes the socio-demographic characteristics of the entire study population.

### Phase I: questionnaire item development

The expert panel devised an initial list of 32 questions. After content validity assessment, only 25 questions were retained since they had CVIs of at least 0.86 (Table 3). Question #7 was removed because the panel experts agreed that sunlight exposure during rainy weather is minimal; questions #21 and #22 were considered redundant since question #2 already inquired regarding clothing and body part exposure; question #24 was removed since wearing sunglasses was considered more for protection from the sun’s glare; question #27 was also considered redundant since question #10 already inquired about the respondent’s usual mode of transport; and questions #31 and #32 were removed since the use of sunlamps and sunbeds is not common among Filipinos.

### Phase II: forward-translation and back-translation

During the translation process, words like “jogging,” “calcium,” “vitamin D,” “multivitamins,” “sunburn,” “allergy,” “heat stroke,” and “sunscreen” were retained in English, as these were deemed familiar terms for ordinary Filipinos. No significant disparities between the English and Filipino (Tagalog) versions were detected by the independent bilingual translators during translation and back-translation.

### Phase III: results of pretest

Pretesting of the prefinal Filipino (Tagalog) SEQ was conducted with 75 participants. The average time needed to complete the 25-item questionnaire was 15 minutes (range, 5-30 minutes). In general, the respondents found the questionnaire to be comprehensible with acceptable length and arrangement. No questions were considered sensitive, biased, or threatening during cognitive debriefing. For questions #17-#19, the phrase “posibilidad ng” (“possibility of”) was added to the beginning of each question to emphasize the risk of sunlight exposure. For questions #23-#25, many respondents preferred the term “sunblock” to “sunscreen.” However, “sunscreen” was retained, as it affords protection from the entire ultraviolet range, as opposed to “sunblock,” which only affords protection from UVB. Furthermore, several guidelines, including those of the US Food and Drug Administration, do not recommend the word “sunblock,” as it may falsely overemphasize a product’s efficacy [16].

### Phase IV: results of construct validity test and reliability test

The final questionnaire was administered to the entire sample of 260 participants. There were no dropouts; all participants completed both the first and second rounds of testing. The mean age was 41.54 years, with a mean duration of urban living of 28.77 months. The majority of the respondents were day shift (79.6%) and indoor (62.7%) workers. There was no significant difference in the mean time needed to complete the first (6.78 minutes) and the second (6.61 minutes) test (Table 2).

Factor analysis yielded 3 principal component factors corresponding to the different questionnaire domains. Items that possessed similar factor loading values were grouped under a particular domain. The 3 domains were labeled: (1) intensity of sunlight exposure (containing questions #1-#7); (2) factors affecting sunlight exposure (containing questions #8-#19); and (3) sun protection practices (containing questions #20-#25). Table 4 shows the 3 domains of the SEQ and the factor loading values of the items in each domain.

The internal consistency assessment yielded an overall Cronbach alpha coefficient of 0.80, indicating that the questionnaire generally showed internal consistency. The 3 domains were internally consistent on their own as well, with coefficient values of 0.74, 0.71, and 0.72, respectively. Similarly, the paired *t*-test yielded no statistically significant differences between the responses obtained in the first and second rounds of testing for either the entire questionnaire or each of its domains, indicating satisfactory test-retest reliability (Table 5).

## DISCUSSION

This is the first SEQ developed and validated for use in an urban adult Filipino population. The questionnaire was designed to assess the intensity of sunlight exposure, the various factors affecting sunlight exposure, and the different sunlight protection practices utilized by urban adult Filipinos.

To ensure adequate representativeness of the sample, our sampling frame took into account age, sex, educational attainment, work shift and location, and economic status. Although it could be argued that elderly respondents should comprise a greater proportion of the sample (given that the consequences of VDD are especially strongly felt in this population), our aim was to create a more even distribution of respondents across the entire adult lifespan to maximize the questionnaire’s applicability [17]. The respondents’ locations within Metro Manila were not part of the sampling frame, since each of the Philippine capital’s 17 component cities are topographically similar, and hence there were no expected significant differences in sunlight exposure. The Köppen climate classification lists Metro Manila as having a uniformly tropical wet and dry climate [18].

The questionnaire development process drew on the existing instruments of Cargill et al. [9] in Australia, Hanwell et al. [8] in Italy, Humayun et al. [6] in Pakistan, and Wu et al. [7] in Hong Kong. Although the questionnaires served as important references, no questions were directly taken from any of these instruments, as they were all developed in countries of a different ethnicity, geography, and climate compared to the Philippines. Hence, we utilized a separate conceptual framework that explored additional aspects of sunlight exposure in Filipinos that may not have been covered in the existing questionnaires [11]. A unique feature of our questionnaire is the inclusion of questions pertaining to the perceived risks and benefits of sunlight exposure, which are significant determinants of an individual’s sunlight exposure practices. We also added questions pertaining to the influences of other people and mass media on sunlight exposure, given the strong kinship and social ties among Filipinos and the widespread use of technology by urban residents [19]. A current disadvantage of the questionnaire is the lack of a validated scoring system and the lack of correlation with established gold standard measurements, the latter of which will be addressed in the next phase of the study.

In the construction of questionnaire items, we utilized the Likert scale, the most widely-used approach to scaling responses in questionnaire research. Unlike simple close-ended questions, the Likert scale has the ability to specify levels of agreement or disagreement in a symmetric fashion, capturing the range of intensity of feelings for a given item, which is then simplified as the sum of the questionnaire items [20]. Questionnaires in Likert scale format are also easy to use and allow more variables in a study because the format enables respondents to answer more questions in the same time required to answer fewer open-ended questions [21,22]. While we retained the same choices for many questions (“never,” “rarely,” “often,” and “always”), the choices for other questions were crafted to reflect a similarly symmetric degree of sun exposure. This was especially true for questions involving the Fitzpatrick skin classification, body part exposure, and temporal exposure.

During the translation and back-translation process, the independent bilingual translators decided to retain several words in English. This is due to the fact that these particular words are considered familiar terms for ordinary Filipinos. In the 2010 Test of English as a Foreign Language, the Philippines ranked 35th out of 163 countries worldwide, and ranked second-best in Asia after Singapore [23].

The questionnaire was assessed using content validity, construct validity, and reliability. Content validity, which refers to the representativeness or relevance of the questionnaire content, was assessed individually by the members of an expert panel [7]. These members were chosen from diverse disciplines to ensure a holistic clinical and psychosocial evaluation of the questionnaire items. While majority of the items possessed sufficient content validity, those that were removed were mostly either redundant or found to be non-contributory to sunlight exposure evaluation. Others (such as the use of sunlamps or sunbeds) were deemed not applicable to Filipino culture.

Our questionnaire also possessed satisfactory construct validity. The 3 domains extracted after factor analysis corresponded well with the themes identified during creation of the conceptual framework. Specifically, the first 2 domains corresponded to the influences and perceived benefits and risks of sunlight exposure. The third domain also corresponded to perceived risks, as an increased awareness of these risks leads to an increased usage of sun protection practices (Figure 1). Furthermore, the factor analysis results also fulfilled the rule of having a minimum of 5 questions per domain to enable psychometric testing, with 7 questions in the first domain, 12 questions in the second domain, and 6 questions in the third domain [24]. Reliability, meanwhile, was likewise sufficient in terms of both internal consistency and test-retest reliability. For the latter, the decision to administer the questionnaire 2 weeks apart was made because that time frame was long enough for the participants to not remember their responses from the first test, while being short enough to not allow significant physiological changes to occur. There was also no significant difference in the time needed to complete the first and second rounds of testing, attesting to the questionnaire’s consistency in ease of administration.

This study serves as part of a larger project that will eventually involve concurrent and criterion validity assessment of the questionnaire results with established objective parameters, such as dosimetry and serum 25-OHD levels. We also recommend future studies investigating the applicability of the questionnaire to a wider population, particularly rural and other urban areas in the Philippines, in addition to other Southeast Asian and tropical countries of similar ethnicity and geographical latitude.

In conclusion, this study showed that a linguistically and culturally appropriate SEQ possessed sufficient content validity, construct validity, and reliability to assess sunlight exposure among urban adult Filipinos in Metro Manila. The questionnaire results can be eventually applied to evaluate associations with serum 25-OHD levels.

## Figures and Tables

**Table 1.** Sampling frame for the study

	Working age (19-60 yr)			Elderly/retirees (>60 yr)
	Age (yr)	Professional, mostly indoor work	Some education, mostly outdoor work	
Male	19-30	15	15	10
	31-40	15	15	
	41-50	15	15	
	51-60	15	15	
Female	19-30	15	15	10
	31-40	15	15	
	41-50	15	15	
	51-60	15	15	

**Table 2.** Distribution of respondents according to socio-demographic characteristics (n=260)

Characteristics	n (%)
Age (mean±SD, yr)	41.54±13.60
Sex	
Female	130 (50.0)
Male	130 (50.0)
Duration of urban living in months (mean±SD)	28.77±18.60
Educational attainment	
Elementary graduate or less	7 (2.7)
High school graduate or less	63 (24.2)
Some college/vocational course	63 (24.2)
College graduate	127 (48.8)
Type of work	
Day shift	207 (79.6)
Night shift	34 (13.1)
Unemployed	19 (7.3)
Location of work	
Indoor	163 (62.7)
Outdoor	97 (37.3)
Household income (PHP/mo)^1^	
≥10,000^1^	176 (67.7)
<10,000^1^	84 (32.3)
Time needed to complete test in minute (mean±SD)	
1st test	6.78±5.40
2nd test	6.61±2.40

SD, standard deviation; PHP, Philippine peso.

1 PHP=US$ 0.019.

**Table 3.** CVI values for items in the Filipino SEQ

No.	Question	CVI
1	How do you describe your skin when it is exposed to the sun?	1.00
2	What part of your body is usually exposed to the sun?	1.00
3	How long do you usually spend under the sun on a weekday?	1.00
4	How long do you usually spend under the sun on a weekend?	1.00
5	How long do you usually spend under the sun during sunny weather?	1.00
6	How long do you usually spend under the sun during cloudy weather?	1.00
7	How long do you usually spend under the sun during rainy weather?	0.67^1^
8	What time of the day are you usually exposed to the sun?	1.00
9	How often do you go out in the sun due to work or daily routine?	1.00
10	How often do you walk or use public transport to do the above activities?	1.00
11	How often do you engage in outdoor activities such as jogging, cycling, and swimming?	1.00
12	How often do you take calcium with vitamin D or multivitamins?	1.00
13	How likely are you to be exposed to the sun to get stronger bones and better health?	1.00
14	How likely are you to be exposed to the sun to get happier and livelier?	1.00
15	How likely are you to be exposed to the sun to get more beautiful skin?	1.00
16	How likely are you to avoid sun exposure due to the influence of family, friends, and coworkers?	1.00
17	How likely are you to avoid sun exposure due to the influence of TV, radio, and internet?	1.00
18	How likely are you to avoid sun exposure due to sunburn, skin cancer, skin allergy, and rashes?	1.00
19	How likely are you to avoid sun exposure due to heat stroke, hypertension, and dizziness?	1.00
20	How likely are you to avoid sun exposure due to sweating and fear of darker skin?	1.00
21	When going out in the sun, how often do you wear long sleeves?	0.33^1^
22	When going out in the sun, how often do you wear long pants?	0.33^1^
23	When going out in the sun, how often do you wear a hat?	1.00
24	When going out in the sun, how often do you wear sunglasses?	0.17^1^
25	When going out in the sun, how often do you use an umbrella?	1.00
26	When going out in the sun, how often do you walk under the shade?	1.00
27	When going out in the sun, how often do you use transportation with closed windows?	0.17^1^
28	When going out in the sun, how often do you use sunscreen containing at least SPF 30?	1.00
29	When do you usually apply sunscreen?	1.00
30	How much sunscreen do you usually apply?	1.00
31	How often do you use a sunlamp?	0.00^1^
32	How often do you use a sunbed?	0.00^1^

CVI, content validity index; SEQ, sunlight exposure questionnaire; SPF, sun protection factor.

1 Eventually omitted from the questionnaire due to CVI < 0.86.

**Table 4.** Factor loadings by factor analysis of items of the Filipino SEQ

Item no.		Factor		
		1	2	3
1	How do you describe your skin when it is exposed to the sun?	-0.25	0.10	-0.06
2	What part of your body is usually exposed to the sun?	0.20	0.20	0.01
3	How long do you usually spend under the sun on a weekday?	0.27	0.57	-0.16
4	How long do you usually spend under the sun on a weekend?	0.22	0.59	-0.15
5	How long do you usually spend under the sun during sunny weather?	0.31	0.47	-0.15
6	How long do you usually spend under the sun during cloudy weather?	0.24	0.26	-0.12
7	What time of the day are you usually exposed to the sun?	0.28	0.31	0.17
8	How often do you go out in the sun due to work or daily routine?	0.43	0.16	-0.24
9	How often do you walk or use public transport to do the above activities?	0.19	0.17	0.12
10	How often do you engage in outdoor activities such as jogging, cycling, and swimming?	0.18	0.13	-0.12
11	How often do you take calcium with vitamin D or multivitamins?	0.05	-0.03	-0.23
12	How likely are you to be exposed to the sun to get stronger bones and better health?	0.62	0.10	-0.61
13	How likely are you to be exposed to the sun to get happier and livelier?	0.59	-0.11	-0.59
14	How likely are you to be exposed to the sun to get more beautiful skin?	0.43	-0.13	-0.48
15	How likely are you to avoid sun exposure due to the influence of family, friends, and coworkers?	0.23	0.10	0.17
16	How likely are you to avoid sun exposure due to the influence of TV, radio, and internet?	0.19	0.14	0.17
17	How likely are you to avoid sun exposure due to sunburn, skin cancer, skin allergy, and rashes?	0.64	-0.57	0.18
18	How likely are you to avoid sun exposure due to heat stroke, hypertension, and dizziness?	0.61	-0.53	0.05
19	How likely are you to avoid sun exposure due to sweating and fear of darker skin?	0.46	-0.09	0.13
20	When going out in the sun, how often do you wear a hat?	-0.13	-0.05	0.26
21	When going out in the sun, how often do you use an umbrella?	0.22	0.10	0.43
22	When going out in the sun, how often do you walk under the shade?	0.24	0.03	0.33
23	When going out in the sun, how often do you use sunscreen containing at least SPF 30?	0.35	0.26	0.65
24	When do you usually apply sunscreen?	0.44	0.27	0.63
25	How much sunscreen do you usually apply?	0.39	0.31	0.65

SEQ, sunlight exposure questionnaire; SPF, sun protection factor.

**Table 5.** Per-domain test-retest reliability of the Filipino SEQ

Sunlight exposure domains	First test	Retest	p-value
Overall for questionnaire	2.39±0.36	2.40±0.37	0.73
Domain 1 (intensity of sunlight exposure)	1.92±0.49	1.90±0.45	0.54
Domain 2 (factors affecting sunlight exposure)	2.47±0.43	2.50±0.45	0.34
Domain 3 (sun protection methods)	2.77±0.65	2.76±0.64	0.96

Values are presented as mean±standard deviation. SEQ, sunlight exposure questionnaire.
